# “IT’S NOT THE AGE, IT’S THE APTITUDE: FACTORS INFLUENCING ADOPTION OF DIGITAL HEALTH APP AMONG OLDER ADULTS”

**DOI:** 10.1093/geroni/igae098.4098

**Published:** 2024-12-31

**Authors:** Yosefa Birati, Hod Orkibi, Lola Kolpina, Anna Zisberg

**Affiliations:** University of Haifa, Haifa, Hefa, Israel; University of Haifa, Haifa, Hefa, Israel; University of Haifa, Haifa, Hefa, Israel; University of Haifa, Haifa, Hefa, Israel

## Abstract

The rapid growth of digital health services has established mobile health (mHealth) apps as a key communication channel between healthcare providers and patients in primary care. These apps offer features such as appointment scheduling, access to medical records, and administrative task management. This study examines how digital health literacy, perceived ease of use, and perceived usefulness of mHealth apps explain mHealth apps and online services usage among older adults. Our study involved 179 community-dwelling older adults (mean age 73.5±5.05). The sample was relatively well-educated, with 33.2% holding a bachelor’s degree and 31.5% having a master’s or higher degree. Notably, 80% of participants reported high to very high confidence in using mHealth apps. Multiple regression analysis showed that age and health status alone explained only 4% of the variance in mHealth app use. However, when accounting for digital health literacy (β =.228, p <.001), ease of use (β =.369, p <.001), and usefulness (β =.283, p <.001) the model accounted for 58.7% of the variance (ΔR² =.493, p <.001). These findings underscore the critical importance of addressing digital health literacy and usability challenges among older adults. By focusing on these factors over age alone, we can enhance the design and personalization of mHealth apps and facilitate independent engagement with digital health services among the older adult population.

